# Naturally Occurring Calanolides: Occurrence, Biosynthesis, and Pharmacological Properties Including Therapeutic Potential

**DOI:** 10.3390/molecules25214983

**Published:** 2020-10-28

**Authors:** Lutfun Nahar, Anupam Das Talukdar, Deepa Nath, Sushmita Nath, Aman Mehan, Fyaz M. D. Ismail, Satyajit D. Sarker

**Affiliations:** 1 Laboratory of Growth Regulators, Institute of Experimental Botany ASCR & Palacký University, Šlechtitelů 27, 78371 Olomouc, Czech Republic; 2 Department of Life Science and Bioinformatics, Assam University, Silchar, Assam 788011, India; adtddt@gmail.com; 3 Department of Botany, Gurucharan College, Silchar, Assam 788004, India; deepa.nath@gmail.com; 4 Centre for Natural Products Discovery, School of Pharmacy and Biomolecular Sciences, Liverpool John Moores University, James Parsons Building, Byrom Street, Liverpool L3 3AF, UK; sushmitanath84@gmail.com (S.N.); fyaz.ismail@gmail.com (F.M.D.); 5 School of Clinical Medicine, University of Cambridge, Cambridge CB2 OSP, UK; ahm41@cam.ac.uk

**Keywords:** calanolides, pseudocalanolides, calanolide A, *Calophyllum*, Calophyllaceae, anti-HIV, reverse transcriptase, non-nucleoside reverse transcriptase inhibitors (NNRTIs)

## Abstract

Calanolides are tetracyclic 4-substituted dipyranocoumarins. Calanolide A, isolated from the leaves and twigs of *Calophyllum lanigerum* var.* austrocoriaceum* (Whitmore) P. F. Stevens, is the first member of this group of compounds with anti-HIV-1 activity mediated by reverse transcriptase inhibition. Calanolides are classified pharmacologically as non-nucleoside reverse transcriptase inhibitors (NNRTI). There are at least 15 naturally occurring calanolides distributed mainly within the genus *Calophyllum,* but some of them are also present in the genus *Clausena*. Besides significant anti-HIV properties, which have been exploited towards potential development of new NNRTIs for anti-HIV therapy, calanolides have also been found to possess anticancer, antimicrobial and antiparasitic potential. This review article provides a comprehensive update on all aspects of naturally occurring calanolides, including their chemistry, natural occurrence, biosynthesis, pharmacological and toxicological aspects including mechanism of action and structure activity relationships, pharmacokinetics, therapeutic potentials and available patents.

## 1. Introduction

**Figure 1:**
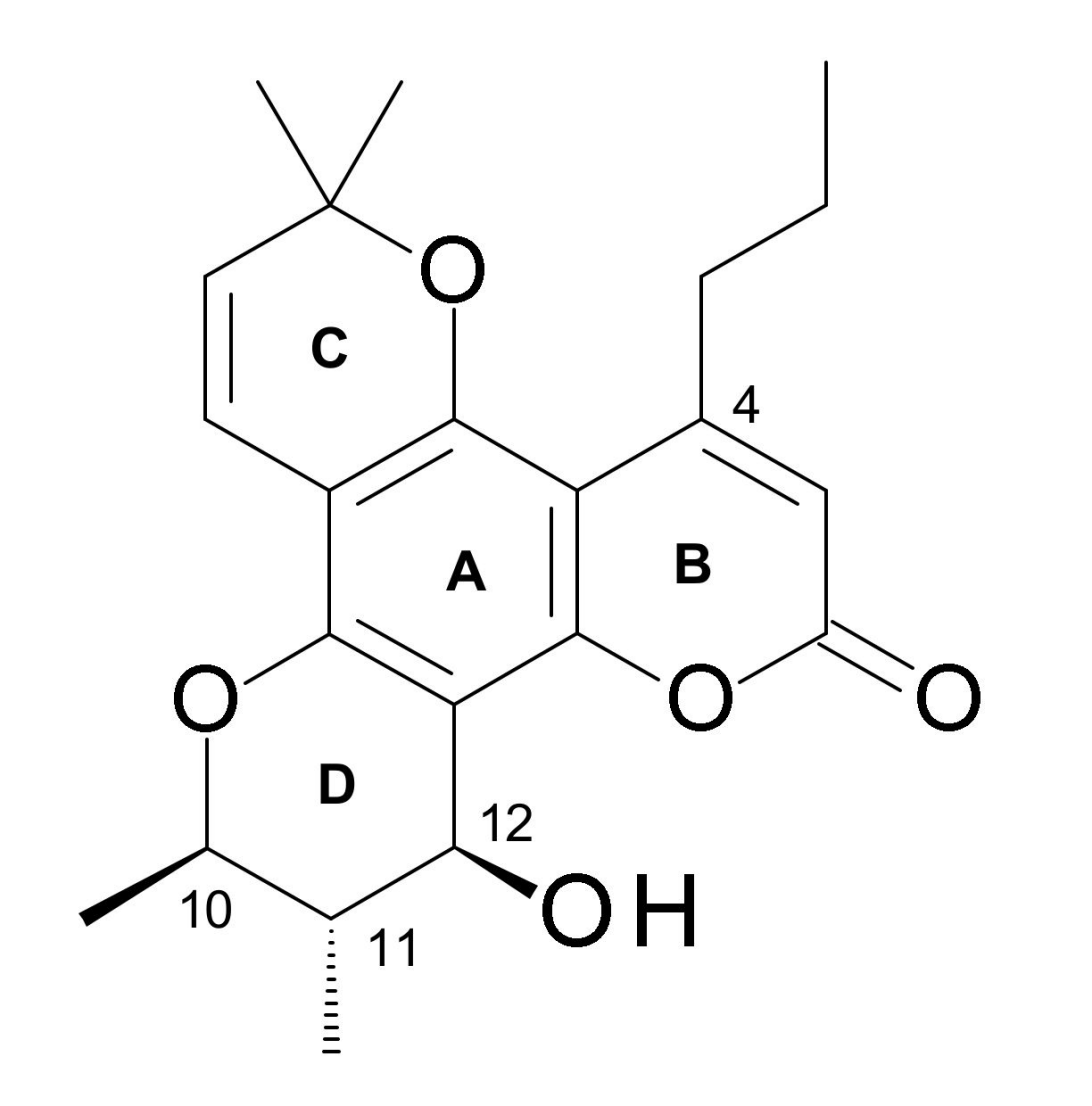
Rings A, B, C and D, and carbon numbering in (+)-calanolide A (**1**).

**Figure 2:**
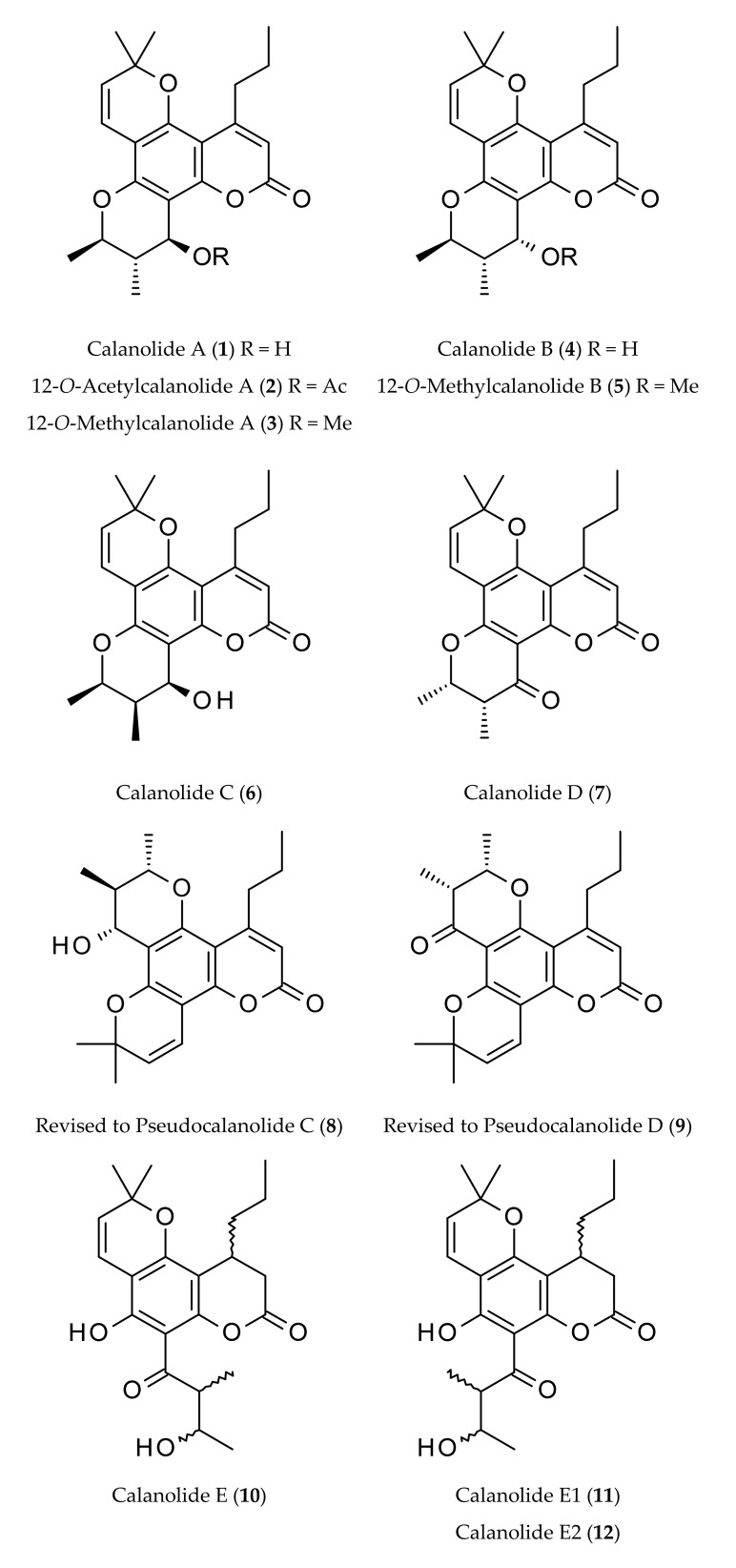

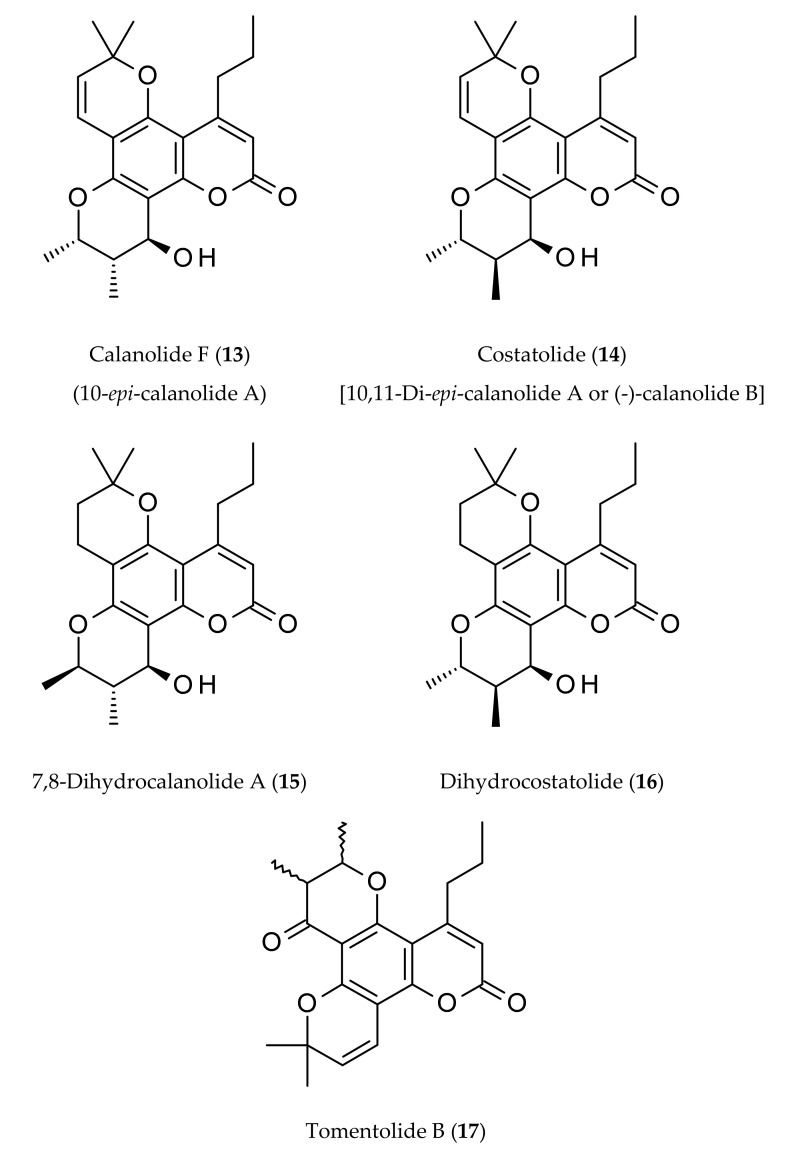
Naturally occurring calanolides.

Calanolides are tetracyclic 4-substituted dipyranocoumarins, and their C-ring contains a *gem*-dimethyl group (Figure 1), e.g., (+)-calanolide A (**1**), (−)-calanolide B (costatolide) (**14**) (Figure 2). The discovery of calanolides from the leaves and twigs of the tree *Calophyllum lanigerum var. austrocoriaceum* (Whitmore) P. F. Stevens, collected from Sarawak, Malaysia in 1987 happened during one of the largest anti-HIV screening programs conducted by the National Cancer Institute (NCI) during 1987–1996. In that program, over 30,000 plant extracts were screened utilizing an in vitro cell-based anti-HIV screen that could determine the degree of HIV-1 replication in treated infected lymphoblastic cells versus that in treated uninfected control cells [1,2]. Calanolide A (**1**) (Figure 1), which can be described as a 11,12-dihydro-2*H*,6*H*,10*H*-dipyrano[2,3-f:2′,3′-h]chromen-2-one substituted by a hydroxyl (–OH) group at C-12, methyl groups at positions 6, 6, 10 and 11 and a propyl group at C-4 (the 10*R*,11*S*,12*S* stereoisomer), was isolated as the first member of anti-HIV compounds, calanolides, as a potential novel therapeutic option for the treatment of HIV infections. However, a subsequent attempt to recollect this plant sample failed and the collection of other specimens of the same species (not necessarily the same variety), afforded only a negligible amount of calanolide A (**1**). In fact, calanolides are among the first plant-based compounds to demonstrate potential anti-HIV-1 activity. Later, an extract of the latex of *C. teysmanii* showed significant anti-HIV activity in the screening, but the major active compound was (−)-calanolide B (**14**, also known as costatolide), regrettably not calanolide A (**1**) (Figure 2). The anti-HIV activity of (−)-calanolide B (**14**) was less potent than that of calanolide A (**1**), possibly because of difference in stereochemistry at the chiral centers. To date calanolides A-F and some of their methyl, acetyl and dihydro derivatives have been reported mainly from various *Calophyllum* species (Figure 2). Among these, the structures of calanolides C (**6**) and D (**7**), as reported initially by Kashman et al. [1] from *C. lanigerum*, were revised and renamed as pseudocalanolides C (**8**) and D (**9**) [3] (Figure 2). However, the true calanolides C (**6**) and D (**7**) were later reported from *C. brasiliense* Cambess. [4,5,6]. 

The first isolation of calanolides from *C. lanigerum var. austrocoriaceum,* involved multiple steps, starting with the extraction of dried fruits and twigs of this plant with a 1:1 mixture of dichloromethane and methanol, followed by a sequential solvent partitioning process involving various solvents. The *n*-hexane and CCl_4_ fractions emerged as the active fractions [1]. Repeated vacuum liquid chromatography (VLC) on silica gel, eluting with a mixture of *n*-hexane and ethyl acetate afforded crude calanolides, which were further purified by HPLC, employing normal phase for calanolide A (**1**), calanolide B (**4**) and pseudocalanolide D (**9**) [reported incorrectly as calanolide D (**7**)], while reversed-phase for 12-acetoxycalanolide A (**2**), 12-methoxycalanolide A (**3**), 12-methoxycalanolide B (**5**), pseudocalanolide C (**8**) [reported as calanolide C (**6**)] and calanolide E (**10**). The structures of these compounds were determined by a combination of UV, IR, NMR and MS spectroscopic methods, and all spectroscopic data were published [1]. The absolute stereochemistry of calanolides A (**1**) and B (**4**) was confirmed by a modified Mosher′s method.

There is a review [7] and a book chapter on calanolides [8], published about six years ago, that mainly cover anti-HIV activity, and the literature published until early 2014. This present review is not on the genus *Calophyllum*, the family Calophyllaceae or pyranocoumarins a such, but it exclusively focuses on various aspects of naturally occurring calanolides. This review is significantly different from any other previous articles on calanolides in its approach and coverage, and is a comprehensive update on naturally occurring calanolides, encompassing their chemistry, natural occurrence, biosynthesis, pharmacological and toxicological aspects including mechanism of action and structure activity relationships, pharmacokinetics, therapeutic potentials and available patents.

## 2. Occurrence

Calanolides, calanolide A (**1**) being the first member of these 4-substituted pyranocoumarins isolated from *C. lanigerum var. austrocoriaceum*, are almost exclusively distributed within the genus *Calophyllum* L., which comprises a large group of ca. 200 species of tropical trees distributed in the Indo-Pacific region, but was also reported from one species (*Clausena excavate* Brum. f.) of the closely related genus *Clausena* [9,10,11,12] (Table 1). Calanolide A (**1**) and other calanolides were subsequently isolated from other *Calophyllum* species, e.g., *C. brasiliense* Cambess. [4,13,14], *C. inophyllum* L. [6], *C. teysmanii* Miq. [2] and *C. wallichianum* Planch. & Triana [15]. In a chemotaxonomic study on the *Calophyllum* species, the presence of calanolides was detected in the extracts of *C. inophyllum*, *C. lanigerum* var. *austrocoriaceum*, *C. mole* King, *C. nodosum*, aff. *Pervillei* Vesque., *C. soulattri* Burm. f., *C. tacamahaca* Willd. and *C. teysmanii* [9] (Table 1). 

Bernabe-Antonio et al. [5] reported the production of calanolides in a callus culture of *C. brasiliense*, where different concentrations and combinations of plant growth regulators were tested in leaf and seed explants to establish callus cultures capable of producing calanolides. Higher calanolides B (**4**) and C (**6**) production was observed in calluses from seed explants than those developed from leaves. In continuation of the search for new natural anti-HIV compounds, and at the same time to find new botanical sources of calanolides, McKee et al. [20] purified calanolide E2 (**12**), and calanolide F (**13**) from the extracts of *C. lanigerum var. austrocoriaceum* and *C. teysmanii var. inophylloide* (King.) P. F. Stevens. Later, costatolide (**14**), also known as (−)-calanolide B, was reported as an anti-HIV compound present in *C. cerasiferum* Vesque and *C. inophyllum* L. [24]. Calanolides A (**1**), and C (**6**), and costatolide (**14**) were isolated from the leaves of *C. brasiliense*, and their anti-HIV potential was evaluated [4].

## 3. Biosynthesis

**Scheme 1:**
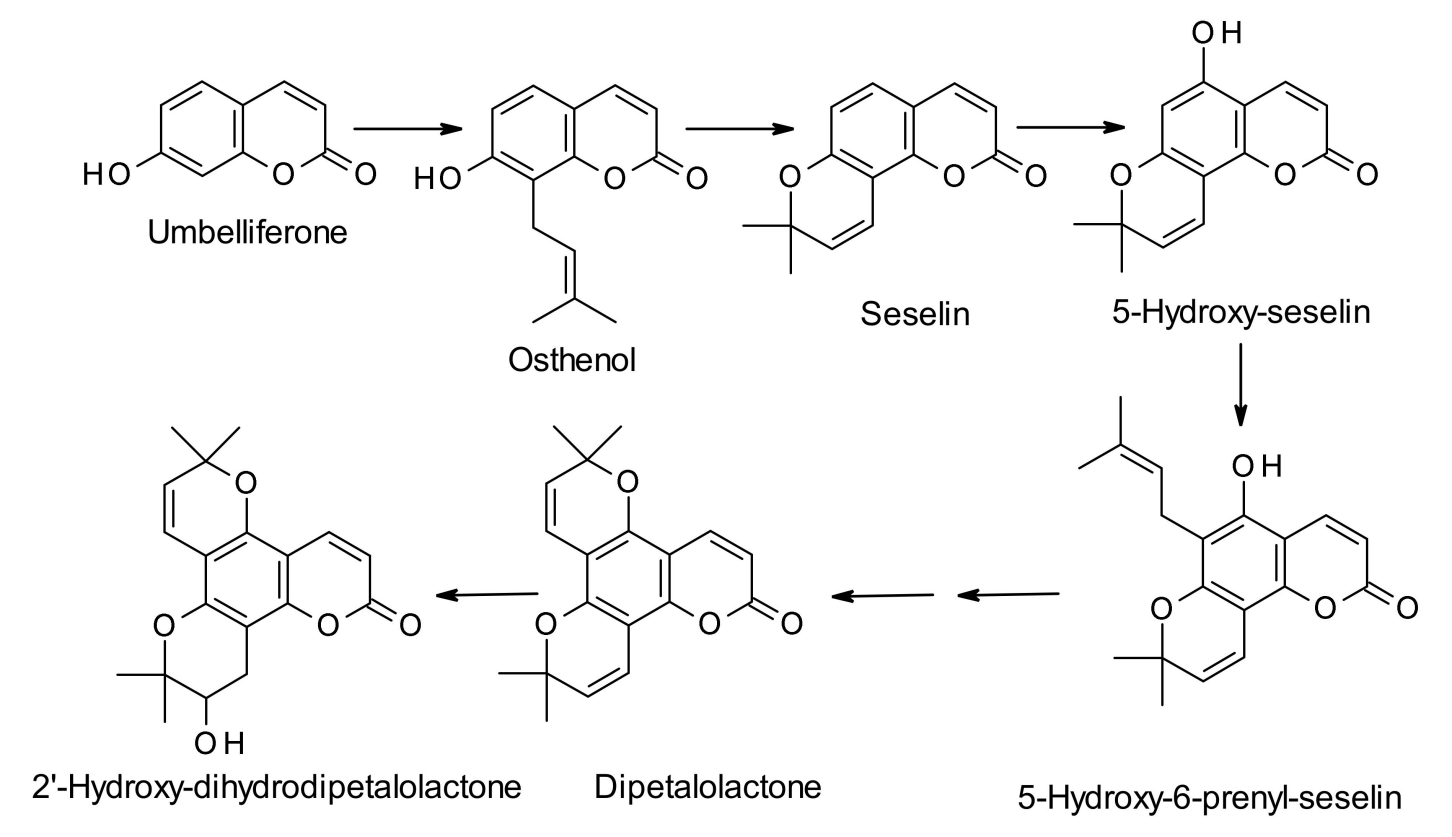
Plausible biosynthetic route to 2′-hydroxydihydrodipetalolactone from umbelliferone *via* formation of 5-hydroxy-6-prenylseselin.

**Scheme 2:**
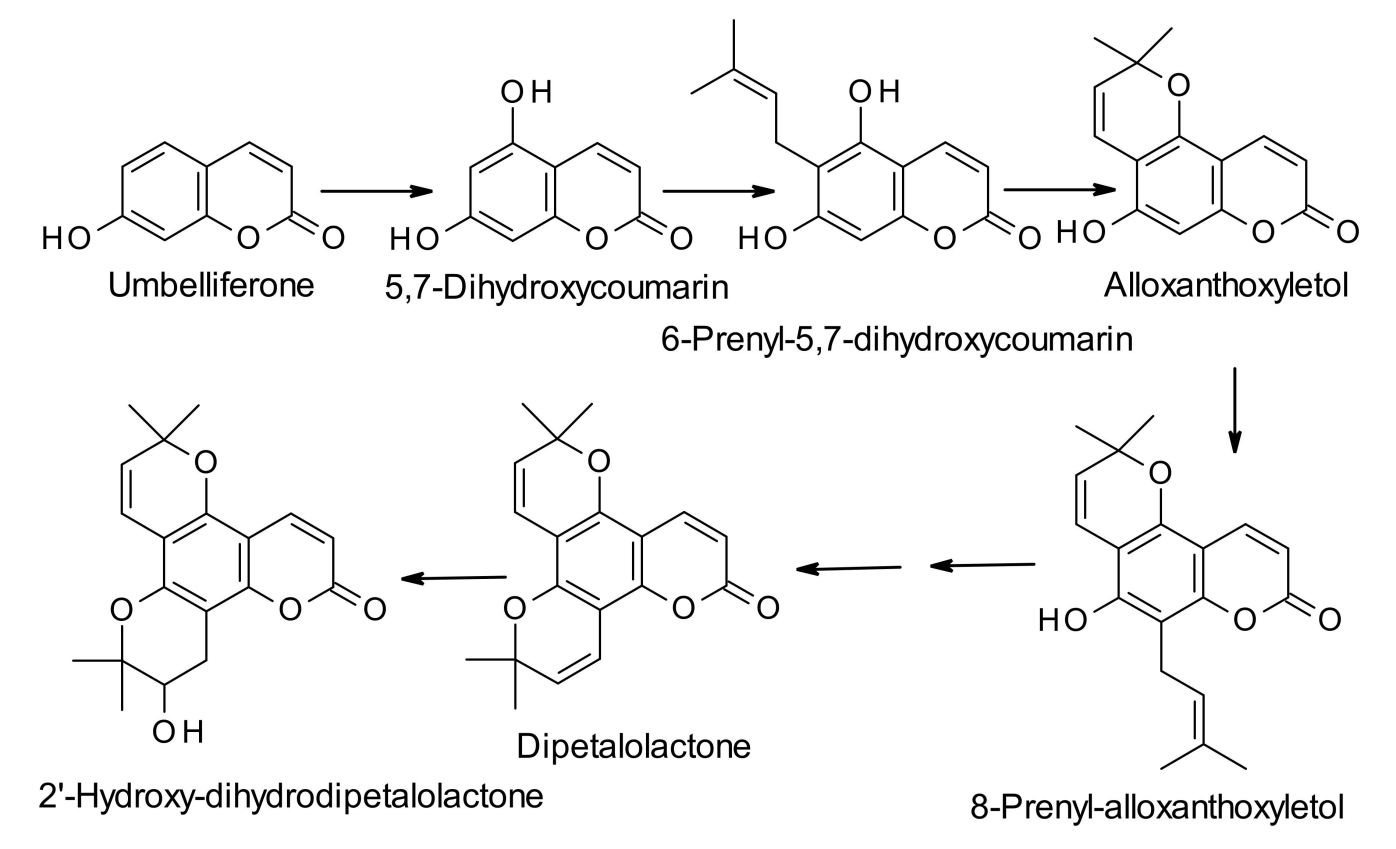
Plausible biosynthetic route to 2′-hydroxydihydrodipetalolactone from umbelliferone *via* formation of 8-prenylalloxanthoxyletol.

**Scheme 3:**
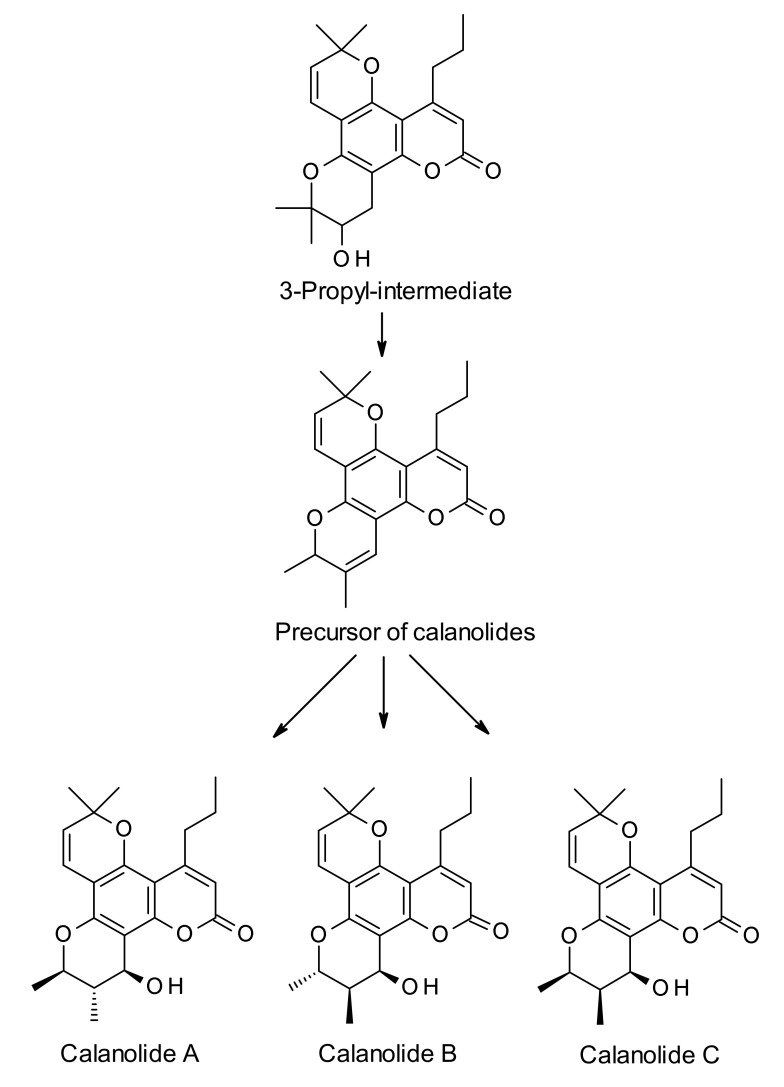
Plausible biosynthetic route to calanolides A (**1**), B (**4**) and C (**6**) from the intermediate, 2′-hydroxydihydrodipetalolactone.

Calanolides are biosynthesized from the parent simple coumarin 7-hydroxycoumarin, also known as umbelliferone (Scheme 1, Scheme 2 and Scheme 3). The biosynthesis of umbelliferone in plants starts from the amino acid L-phenylalanine, and proceeds through the formation of *trans*-cinnamic acid, *p*-coumaric acid, 2-hydroxy-*p*-coumaric acid, 2-glucosyloxy-*p*-coumaric acid, and 2-glucosyloxy-*p*-*cis*-coumaric acid with the help of various enzymes like cinnamate 4-hydroxylase, 4-coumarate-CoA ligase, 4-coumaroyl 2′-hydroxylase and so on [27]. The biosynthesis of dipetalolactone, a pyranocoumarin, and subsequent conversion to the 3-propyl-intermediate for calanolides may proceed through two routes, one through conversion of umbelliferone to osthenol (Scheme 1), and the other via formation of 5,7-dihdroxycoumarin (Scheme 2). Reactions are generally mediated by p450 monooxygenase and other non-p450 enzymes [28]. 3-Propyl-intermediate is converted to the precursor compound for calanolides A–C (**1, 4** and **6**), utilizing the Wagner-Meerwein rearrangement reaction, and the precursor compound is believed to be converted to calanolides with the help of p450 monooxygenase enzyme (Scheme 3). Published studies on the biosynthesis of calanolides are rather limited and only two publications are available on this topic to date [28,29]. Therefore, detailed knowledge of specific enzymes involved in the biosynthesis of calanolides is still in its infancy.

In a recent study, the influence of soil nutrients, e.g., Ca^2+^ and K^+^, on the biosynthesis of pharmacologically active calanolides in the seedlings of *C. brasiliense* was studied [29]. It was observed that the use of K^+^ deficient modified Hoagland solution (MHS) could induce a 15, 4.2 and 4.3-fold decrease of calanolides B (**4**), C (**6**), and apetalic acid concentrations in the leaves of the seedlings, respectively. On the other hand, Ca^2+^ deficient MHS could lead to a decrease of 4.3 and 2.4-fold for calanolides B (**4**) and C (**6**), respectively. This study demonstrated that, like many other plant secondary metabolites, the biosynthesis of calanolides, albeit genetically controlled, may also be affected by environmental conditions, e.g., soil nutrients (minerals). 

As genes dictate biosynthesis of secondary metabolites, a study was conducted to identify candidate genes that regulate to the biosynthesis of calanolides in *C. brasiliense* [28]. The unigene dataset constructed in this study could offer an insight for further molecular studies of *C. brasiliense*, particularly for characterizing candidate genes responsible for the biosynthesis of angular and linear pyranocoumarins. The candidate genes, e.g., UN36044, UN28345 and UN34582, identified in the transcriptome of the leaves, stem and roots of *C. brasiliense* might be involved in the biosynthesis of calanolides, which are essentially modified angular pyranocoumarins. Candidate unigenes in the transcriptome dataset were screened using mainly homology-based BLAST and phylogenetic analyses. It is worthy of mention that the BLAST programs are widely used for searching protein and DNA databases for optimizing sequence similarities [30]. For protein comparisons, several definitional, algorithmic and statistical refinements allow substantial decrease in the execution time of the BLAST programs and enhancement of their sensitivity to weak similarities.

## 4. Pharmacological Properties

**Figure 3:**
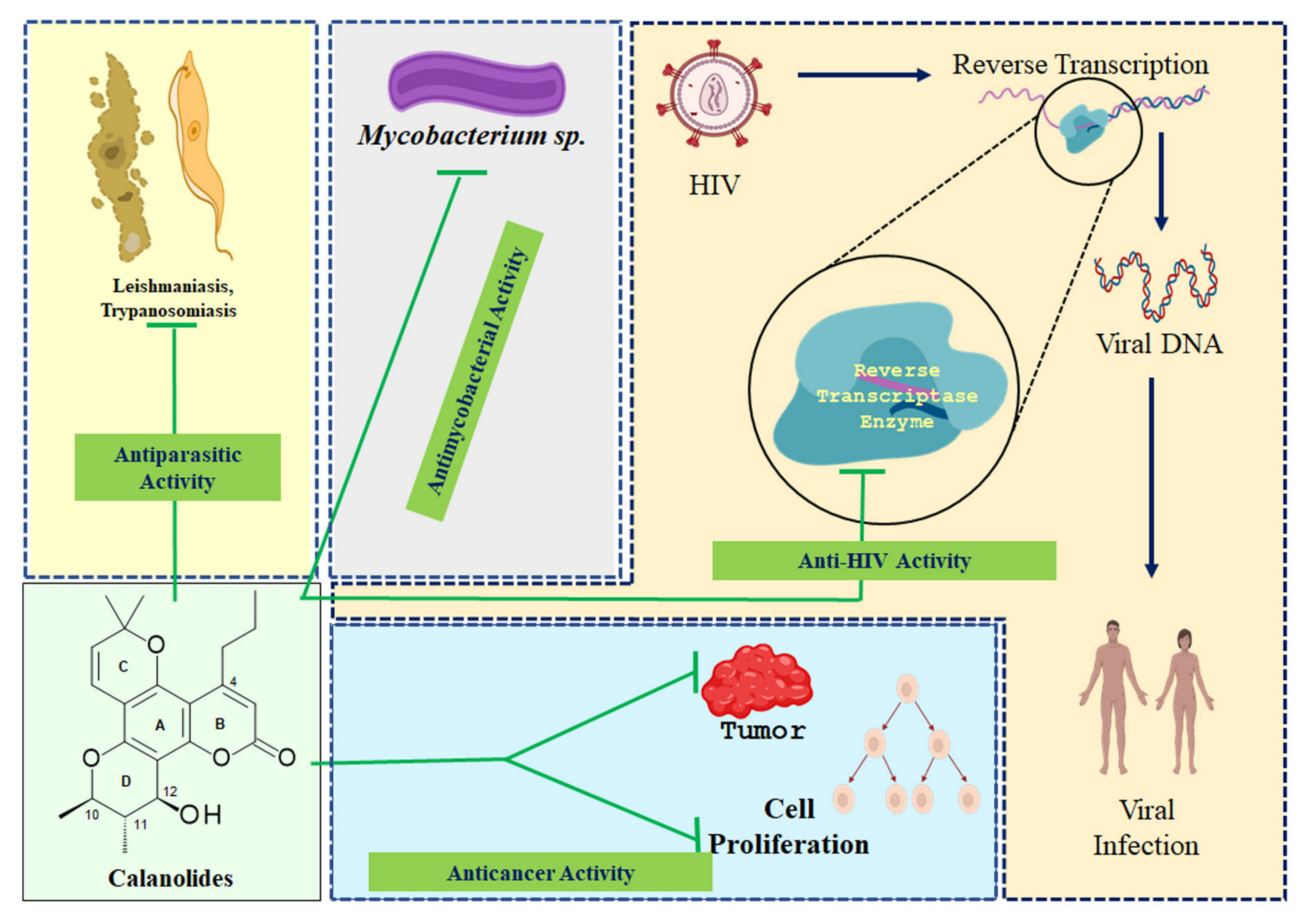
A pictorial summary of pharmacological properties of naturally occurring calanolides.

Although well-known for non-nucleoside reverse transcriptase inhibitory activity offering anti-HIV potential, calanolides have also been shown to possess various other pharmacological properties (Figure 3). The following sub-sections deal with anticancer, anti-HIV, antimycobacterial and antiparasitic activity of naturally occurring calanolides. As much of the published pharmacological studies, both in vitro and in vivo including human trials, on naturally occurring calanolides are about their anti-HIV property, over the years, significant amounts of information have become available on their mechanism of action, structure-activity-relationships, synergistic and/or additive property and their potential in anti-HIV combination therapy, which have been discussed adequately under individual headings within the anti-HIV sub-section. All other pharmacological properties of these compounds as outlined in different publications still require further investigations to establish their realistic therapeutic potential. Also, in silico pharmacological activity and toxicity studies on these pyranocoumarins have just begun to emerge in recent years.

### 4.1. Anticancer Activity

In the later part of 1980s, as a part of the initiative of the United States National Cancer Institute (NCI), plant samples from the Malaysian flora were collected for routine screening for potential cytotoxicity against a collection of cancer cell lines as well as for possible anti-HIV activity. One of the samples, the leaves and twigs of the tree *C. lanigerum* var. *austrocoriaceum*, despite not being active against any of the cancer cell lines tested, showed inhibitory activity of viral replication when tested against HIV-1 virus [31,32]. However, later, calanolide A (**1**) and calanolide C (**6**) were shown to possess antiproliferative or antitumor-promoting property through inhibition of TPA-induced EBV-EA activation in Raji cell lines [13]. The phorbol ester, 12-*O*-tetradecanoylphorbol-13-acetate (TPA) is a potent stimulator of differentiation and apoptosis in myeloid leukemia cells. Calanolide A (**1**) was found to be more active (IC_50_ = 290 mol ratio/32 pmol TPA) than its 10,11-*cis*-isomer, calanolide C (**6**) (IC_50_ = 351 mol ratio/32 pmol TPA). It was inferred that 4-substituted pyranocoumarins like calanolides might possess potential as cancer chemopreventive agents or antitumor-promoters. A recent study with the crude ethanolic extract of the leaves of *C. inophyllum* revealed its potential as a cytotoxic agent (IC_50_ 120 μg/mL) against the breast cancer cell line MCF-7 [33]; it was also found to possess antiproliferative and apoptotic properties. However, no definitive proof was provided to establish which of the secondary metabolites biosynthesized by this plant, calanolides being one major class, were responsible for the putative anticancer activity. Although not calanolides, a few other 4-substituted coumarins, isolated from *C. brasiliense*, were tested against human leukemia HL-60 cells with some promising results [34], which might highlight the need for more comprehensive studies with all major 4-susbtitued coumarins, including calanolides, to find antileukemia lead compounds for new anticancer drug development. Calanolide A (**1**), isolated from a chloroform extract of *Clausena excavata*, was found to induce toxicity to the cells used in a syncytium assay for anti-HIV activity [10]. 

The efficacy of calanolide A (**1**) in AIDS-associated cancer was evaluated in silico utilizing an integrated approach combining network-based systems biology, molecular docking and molecular dynamics [35]. Molecular targets were screened and only the targets, e.g., HRAS, that are common to HIV and sarcoma, HIV and lymphoma, and HIV and cervical cancer, were utilized in this study. Calanolide A (**1**) was found to form a stable complex with the screened target HRAS, which is a small G protein in the RAS subfamily of the RAS superfamily of small GTPases, and is considered as a proto-oncogene; when mutated, this proto-oncogene has the potential to cause normal cells to become cancerous.

### 4.2. Anti-HIV Activity

Calanolide A (**1**), an anti-HIV non-nucleoside reverse transcriptase inhibitor (NNRTI), paved the way for the discovery and synthesis of a series of 4-substituted angular pyranocoumarins with potential anti-HIV property [1,36]. NNRTIs are a class of anti-HIV drugs that prevent healthy T-cells in the body from becoming infected with HIV. Kashman et al. [1] first reported this new class of anti-HIV agents from the tropical rainforest tree, *C. lanigerum*. Calanolide A (**1**), 12-acetoxycalanolide A (**2**), 12-methoxycalanolide A (**3**), calanolide B (**4**), 12-methoxycalanolide B (**5**), pseudocalanolide C (**8**), pseudocalanolide D (**9**) and calanolide E (**10**) (Figure 2) were isolated through an anti-HIV bioassay-guided isolation. Calanolides A (**1**) and B (**4**) were found to be protective against HIV-1 replication and cytopathicity with EC_50_ values of 0.1 µM and 0.4 µM, respectively. However, both compounds were inactive against HIV-2, which is known as less pathogenic than HIV-1 and mainly found in West African countries. The other compounds showed a low level of anti-HIV-1 activity. This study involving purified bacterial recombinant reverse transcriptases established that the calanolides are indeed HIV-1 specific reverse transcriptase inhibitors. A comparative report on the anti-HIV potentials of calanolide A (**1**), costatolide (**14**) and dihydrocostatolide (**16**) against a series of HIV isolates of different cellular phenotypes was published by Buckheit et al. [26], which clearly demonstrated that calanolide A (**1**) was the best anti-HIV candidate among the three calanolides tested. 

Two analogs of calanolide A (**1**), i.e., costatolide (**14**) and dihydrocostatolide (**16**), were shown to possess anti-HIV property similar to that of calanolide A (**1**) [26] and could be ascribed to the class of NNRTIs. In fresh human cells, costatolide (**14**) and dihydrocostatolide (**16**) could significantly inhibit the low-passage clinical virus strains, including those representative of the various HIV-1 clade strains, syncytium-inducing and non-syncytium-inducing isolates, and T-tropic and monocyte-tropic isolates [26,37]. In continuation of the search for new natural anti-HIV compounds, McKee et al. [20] purified calanolide E2 (**12**), and calanolide F (**13**) from extracts of *C. lanigerum* var. *austrocoriaceum* and *C. teysmanii* var. *inophylloide* (King.) P. F. Stevens, and calanolide E2 (**12**) emerged as one of the most active anti-HIV compounds. Later, costatolide (**14**) was reported as an anti-HIV compounds present in *C. cerasiferum* Vesque and *C. inophyllum* L. [24], while calanolides A (**1**), and C (**6**), and costatolide (**14**), isolated from the leaves of *C. brasiliense*, were shown to possess anti-HIV potential [4]. Comparative anti-HIV activities of some naturally occurring calanolides, e.g., calanolide A (**1**), costatolide (**14**) and dihydrocostatolide (**16**), against various strains of HIV are available in the article by Buckheit, et al. [26].

#### 4.2.1. Activity Against Drug Resistant Strains of HIV-1

Interestingly, calanolide A (**1**) was not only found to be active against standard strains of HIV-1, but it was also active against the resistant strains, eAZT-resistant G-9106 strain of HIV-1 and pyridinone-resistant A17 strain [1,38]. The activity against the pyridinone-resistant A17 strain was of interest as this strain is highly resistant to most of the HIV-1 specific NNRTIs, for example, TIBO, BI-RG-587 and L693,593. Later, it was established that pyranocoumarin **1** could interact with HIV-1 reverse transcriptase within the previously defined common binding site for nonnucleoside inhibitors [38]. An assessment of the inhibition patterns of the chimeric reverse transcriptases containing complementary segments of HIV-1 and HIV-2 reverse transcriptases established that there was a segment between residues 94 and 157 in HIV-1 reverse transcriptase that was crucial for inhibition by calanolide A (**1**) [39]. However, it was assumed that there might be a second segment, essential for specifying susceptibility to the drug, between amino acids 225 and 427 in HIV-1 reverse transcriptase. A couple of years later, it was noted that calanolide A (**1**) was active against virus isolates resistant to 1-[(2-hydroxyethoxy)methyl]-6-(phenylthio)thymine and its derivative, [1-benzyloxymethyl-5-ethyl-6-(alpha-pyridylthio)uracil] [40]. Furthermore, this pyranocoumarin (**1**) showed activity against HIV with the two most common NNRTI-related mutations, K103N and Y181C, and was found to select for a mutation that does not cause cross-resistance with any other NNRTIs under investigation. It was postulated that substitution at codon Y188H of reverse transcriptase could be associated with 30-fold resistance to calanolide A (**1**) in vitro [41]. The compound is essentially inactive against all strains of the less common HIV type 2. It is necessary to carry out appropriate in vivo experimentations, either in animal models or in human clinical trials, to understand the true potential of any putative drug candidate. In vivo anti-HIV activity of (+)-calanolide A (**1**) was assessed in a hollow fibre mouse model [42], and it was observed that this compound could suppress virus replication in two unique, but separate physiologic compartments following oral or parenteral administration. 

Calanolides were found to possess an enhanced antiviral activity against one of the most prevalent NNRTI-resistant viruses that is engendered by the Y181C amino acid change in reverse transcriptase as well as with reverse transcriptases that possess the Y181C change together with AZT-resistant mutations [26,37]. Calanolides could also be active against viruses containing Y181C and K103N dual mutations, which are generally highly resistant to other known non-nucleus reverse transcriptase inhibitors. Anti-HIV activity of naturally occurring calanolides against drug-resistant strains of HIV have made these compounds promising structural templates for new anti-HIV drug development.

#### 4.2.2. Calanolides in Anti-HIV-1 Combination Therapy

For the treatment of HIV infections, use of combination therapy comprising several anti-HIV drugs has become a common practice in recent years. The synergistic effects of calanolide A (**1**), costatolide (**14**) and dihydrocostatolide (**16**) [26] in combination with established anti-HIV drugs, e.g., azidothymidine (AZT), indinavir, nelfinavir and saquinavir, are available in the literature [26]. Synergistic effects were observed in both cultured cells and animal models when calanolides were used in combination with other anti-HIV agents [43]. Both calanolide A (**1**) and costatolide (**14**) were found to be effective in combination therapy for HIV infections [44]; in combination with NNRTIs, costatolide (**14**) could only synergistically inhibit HIV type 1 with UC38, whilst calanolide A (**1**) in combination with one of the NNRTIs helped this drug to retain activity against virus isolates with the single Y181C mutation [26,41,44,45]. 

A combination of (+)-calanolide A (**1**) and nevirapine (marketed under the trade name viramune among others for the treatment and prevention HIV-1 infection) was found to possess an additive to weakly synergistic effect in blocking replication of HIV-1 in an in vitro tissue culture assay [41], indicating the possibility of using (+)-calanolide A (**1**) in anti-HIV-1 combination therapy. In an in vivo study using a hollow fibre mouse model [42], the synergistic potential of (+)-calanolide A (**1**) in combination therapy with AZT, a well-known anti-retroviral medication, was further established. A more comprehensive study on the anti-HIV activity of (+)-calanolide A (**1**) and its analogs, e.g., costatolide (**14**), dihydrocostatolide (**16**) and (+)-12-oxo-calanoldie A, in combination with other inhibitors of HIV-1 replication was published about a decade ago [37,46]. Calanolides were found to display synergistic antiviral interactions with other nucleoside and non-nucleoside reverse transcriptase inhibitors and protease inhibitors. In addition, additive interactions were also observed with calanolides when used with other anti-HIV drugs. It was concluded that the utility of convergent and divergent combination therapies using reverse transcriptase inhibitors and protease inhibitors in combination with (+)-calanolide A (**1**) or one of its analogues could be clinically relevant. Budihas et al. [47] demonstrated significant synergy between β-thujaplicinol and calanolide A (**1**).

#### 4.2.3. Structure-Activity-Relationships (SAR)

Among the naturally occurring calanolides, calanolide A (**1**) is one of the most potent anti-HIV compounds and has been the focus of various studies including the study of its possible mechanism of action, structural modifications, pharmacokinetics and toxicity [9,48,49,50,51]. The structures of naturally occurring calanolides mainly differ in their stereochemistry at various chiral centers (C-10, C-11 and C-12) on the ring D (Figure 1 and Figure 2). McKee et al. [20] reported that calanolide-type compounds with a 12β hydroxyl group (as in compound **1**) generally possess anti-HIV activity. While calanolide A (**1**) and costatolide (**14**) were found to be active, (+)-calanolide C (**6**) was inactive in the in vitro anti-HIV assay [4,24]. The inactivity of (+)-calanolide C (**6**) despite possessing the pharmacophoric ring D, as well as a propyl group on C-4, could be due to the β-*cis* orientation of methyl groups on C-10 and C-11. 

Like any other optically active drug molecules, optical activity plays an important role in the anti-HIV activity of calanolides. It has long been established that (+)-calanolide A (**1**) and (−)-calanolide B (**14**) are potent HIV-1 inhibitors, whilst (−)-calanolide A and (+)-calanolide B (**4**) are inactive against the virus [52]. It should be mentioned here that (+)-calanolide A (**1**) is the natural product, but its enantiomer (−)-calanolide A was prepared from the naturally occurring (−)-costatolide (**14**), isolated from *C. costatum*. Similarly, to establish structure-activity-relationships of calanolides, several analogs of calanolides have been synthesized to date, and tested in anti-HIV assays [53]. Although the synthesis of calanolides and the anti-HIV activity of synthetic calanolide analogs are not within the scope of this review, a few examples are given here in the context of structure-activity-relationships. One of the first attempts in this area was from Galinis et al. [53], where Δ^7,8^ olefinic bonds within (+)-calanolide A (**1**) and (−)-calanolide B (**14**) were reduced, and C-12 hydroxyl group in (−)-calanolide B (**14**) was modified to investigate variations in anti-HIV activity compared to parent calanolides. In this study, none of the 14 derivatives was found to possess superior activity to parent calanolides but revealed some preliminary structure-activity requirements for anti-HIV potencies. Later, in order to identify the structural features of naturally occurring (+)-calanolide A (**1**) necessary for its anti-HIV activity and to prepare synthetic analogues, oxo-derivatives (+)-, (−)- and (±)-12-oxocalanolides, were synthesized and tested in vitro using a biochemical reverse transcriptase inhibition assay for determining anti-HIV activity with a promising outcome [48]. In a review article covering various aspects of anti-HIV 4-substitued coumarins with an alkyl or a phenyl group as the substituent, isolated from the genus *Calophyllum*, summarized that all *trans* configurations (10*R*, 11*S*, 12 *S*), as in (+)-calanolide A (**1**) and (+)-inophyllum B (a 4-phenyl-substituted pyranocoumarin), are essential for the best anti-HIV activity [54]. 

Most of the SAR studies involving calanolides for their anti-HIV activities concentrated on the three chiral centers at C-10, C-11 and C-12 of (+)-calanolide A (**1**) [55,56]. As the number of naturally occurring calanolides are rather limited (calanolides A–F) (Figure 2), the SAR studies were often carried out with natural calanolides as well as their synthetic analogs. Of the diastereomers, compounds containing 10,11¬-*trans*-methylation and 12-(*S*)-OH chirality (Figure 2) displayed the most potent activity with EC_50_ values in between 0.18 and 2.0 μM [55]. It was also observed that either the enantiomers (12-*R*-OH) or epimeric alcohols, e.g., calanolide C (**6**) could not produce any noticeable anti-HIV effect. It could be concluded that the relative stereochemistry at C-10 and C-11 are essential structural features for potent anti-HIV activity of calanolides, and at the same time, the *S* configuration at C-12 as well as the presence of a heteroatom, e.g., O, at C-12 are necessary for anti-HIV effects. 

In order to assess the importance of the presence of 11-methyl functionality on calanolide A for its anti-HIV activity, the activity of the semi-synthetic racemic mixture of 11-demethyl-calanolide A was compared with the anti-HIV activity of its parent compound, (±)-calanolide A [57]. The in vitro HIV-1 reverse transcriptase inhibitory activity of these compounds was determined with the isotope 3H assay, which is a thymidine incorporation assay that often utilizes a strategy wherein a radioactive nucleoside, 3H-thymidine, is incorporated into new strands of chromosomal DNA during mitotic cell division; a scintillation beta-counter is used to measure the radioactivity in DNA recovered from the cells in order to determine the extent of cell division that has occurred in response to a test agent. The cytotoxicity and inhibition of cytopathic effect of (±)-calanolide A and (±)-11-demethyl-calanolide A were studied in HIV-1 IIIB infected MT-4 cell cultures by the MTT staining method. Both compounds inhibited HIV-1 reverse transcriptase in vitro with IC_50_ value of 3.028 μM/L and 3.965 μM/L, respectively, for (±)-11-demethyl-calanolide and (±)-calanolide A. They also inhibited cytopathic effect in HIV-1 IIIB infected MT-4 cell cultures with IC_50_ values of 1.081 and 1.297 μM/L, respectively. The outcome form this study indicated that (±)-11-demethyl-calanolide had a slightly more potent anti-HIV activity than (±)-calanolide A, suggesting the methyl functionality at C-11 in calanolide A (**1**) might not be an essential structural feature for anti-HIV activity. With the help of synthetic analogues a few other structural features that could impact on the anti-HIV activity of calanolides could be identified. Some of those are summarized below:

(i) Δ^11,12^ Olefination diminishes activity.
(ii) A C-12 hetero atom is essential for the activity.
(iii) Relative potencies of C-12 ketone, thiol, azide, amine, and acetylated derivatives suggest stringent spatial and stereochemical requirements around C-12.
(iv) The enantiomers of 12-oxocalanolide A, synthetic intermediates containing one fewer chiral center, still retain anti-HIV potency in the cytopathic assays.
(v) The oxygen substituent can either be in the plane of the aromatic system or possess *S* configuration.
(vi) Optical activity is important. For example, (+)-12-oxocalanolide A and (±)-12-oxocalanolide A have similar (but not same) anti-HIV activity, but (−)-12-oxocalanolide A is much less active.
(vii) The racemic form, for example, (±)-12-oxocalanolide A, is more active than its pure (+)-enantiomer, (+)-12-oxocalanolide A, which suggests a possible synergistic effect in the combination of the two enantiomers.
(viii) Hydrogenation at C-7 and C-8 of calanolides has little effect on the anti-HIV activity, e.g., the dihydro derivatives of calanolides A (**1**) and B (**4**) possess the same activity as the parent calanolides.
(ix) Modifications at C-4 substituent can affect the anti-HIV activity of calanolides. For example, a methyl substituent at C-4 (as in cordatolides), instead of a propyl function as in calanolides reduces the anti-HIV potency.
(x) Both the surface area of the substituted group attached on C-10, S-R3, and the distance between atoms O-13 and X-14 (O, N, S), L, of the calanolide analogues play important roles in determining the inhibitory activity of HIV-1 [56].

With the advent of various modern computational tools and mathematical models, it is now possible to study quantitative structure activity relationships (QSAR) in silico, and to predict the potential of any drug candidates for any therapeutic application [58]. A Caco-2 cell permeability QSAR model has recently been used to study various HIV-1 reverse transcriptase inhibitors, including (+)-calanolides A (**1**) and B (**4**), both of which showed a high degree of permeability [59]. This parallel computational screening method incorporated approaches of intestinal absorption prediction, receptor affinity estimation, inhibitor shape similarity, lipophilicity, and index-based lipophilic efficiency analyses. Calanolide A (**1**), among a few other HIV-1 reverse transcriptase inhibitors, emerged as one of the prioritized hits, as a result of guided prioritization task by the better binding affinity, crystal ligand similarity, permissible log*P* value and top lipophilic ligand efficiency scores.

#### 4.2.4. Mechanism of Action

The evaluation of the activity of (+)-calanolide A (**1**) against reverse transcriptase and nonnucleoside reverse transcriptase inhibitor-resistant viruses and enzyme kinetic studies for reverse transcriptase inhibition suggest that this coumarin possibly interacts with the HIV-1 reverse transcriptase in a fashion mechanistically different from other known NNTRIs. The biochemical mechanism of inhibition of HIV-1 reverse transcriptases by calanolide A (**1**) was studied using two primer systems, ribosomal RNA and homopolymeric rA-dT(12-18) [60]. Calanolide A (**1**) was found to bind near the active site of the enzyme and interfered with dNTP binding; it inhibited HIV-1 reverse transcriptase in a synergistic fashion with nevirapine, further distinguishing it from the general class of NNRTIs. It was also observed that at certain concentrations, this compound could bind HIV-1 reverse transcriptase in a mutually exclusive manner with respect to both the pyrophosphate analog, phosphonoformic acid and the acyclic nucleoside analogue 1-ethoxymethyl-5-ethyl-6-phenylthio-2-thiouracil. It was concluded that calanolide A (**1**) could share some binding domains with both phosphonoformic acid and 1-ethoxymethyl-5-ethyl-6-phenylthio-2-thiouracil. It might interact with reverse transcriptase near both the pyrophosphate binding site and the active site of the enzyme. Later, the same group of researchers studied possible mechanism of action of action of calanolide A (**1**) against the HIV type 1 including a variety of laboratory strains, with EC_50_ values of 0.10–0.17 μM [60]. Calanolide (**1**) could inhibit promonocytotropic and lymphocytotropic isolates from patients in various stages of HIV disease, and drug-resistant strains, and was found to act early in the infection process like the known HIV reverse transcriptase inhibitor 2′,3′-dideoxycytidine. It could selectively inhibit recombinant HIV type 1 reverse transcriptase but not cellular DNA polymerases or HIV type 2 reverse transcriptase. Auwerx et al. [50] studied the possible role of Thr139 in the HIV-1 reverse transcriptase sensitivity to (+)-calanolide A (**1**). As T139I reverse transcriptase proved to be resistant to (+)-calanolide A (**1**), represents a catalytically efficient enzyme, and requires only a single transition point mutation (ACA→ATA) in codon 139 could provide some explanation as to why mutant T139I reverse transcriptase virus strains, but not the other strains containing other amino acid changes at this position, predominantly emerge in cell cultures under (+)-calanolide A (**1**) pressure. 

Calanolides are non-nucleoside reverse transcriptase inhibitors and mediate their inhibitory effect in two different template primer systems: primed ribosomal RNA template, and homopolymeric poly rA-oligoT_12-18_ primer. Calanolide A (**1**) was found to inhibit reverse transcriptase by involving two binding sites, and the action is because of the bi-bi ordered mechanism of reverse transcriptase, requiring primer binding prior to polymerization [55]. Calanolide A (**1**) can bind HIV-1 reverse transcriptase in a mutually exclusive manner with the pyrophosphate analogues phosphoformic acid or 1-ethoxymethyl-5-ethyl-6-phenylthio-2-thiouracil. This indicates that calanolide A (**1**) can interact with reverse transcriptase near the pyrophosphate binding site as well as the active site. Unlike general non-nucleoside reverse transcriptase inhibitors, calanolide A (**1**) appears to be at least partially competitive inhibitor of dNTP binding. Clinical and laboratory assessment on viral load and CD4 count indicated that antiviral effects of calanolide A (**1**) appeared to be dose-dependent and maximized on day 14 or 16. Viral life-cycle studies indicated that calanolide A (**1**) could act early in the infection process, similar to the known HIV reverse transcriptase inhibitor 2′,3′-dideoxycytidine. In enzyme inhibition assays, calanolide A (**1**) could potently and selectively inhibit recombinant HIV type 1 reverse transcriptase but not cellular DNA polymerases or HIV type 2 reverse transcriptase within the concentration range tested.

### 4.3. Antimycobacterial Activity

The antibacterial (against *Bacillus cereus, B. pumilius, B. subtilis*, *Escherichia coli*, *Pseudomonas aeruginosa*, *Salmonella typhi*, *Staphylocossus aureus and Vibrio cholerae*) and antifungal (against *Alternaria tenuissima*, *Aspergillus fumigatus*, *Aspergillus niger*, *Candida albicans and Candida tropicalis*) properties of *Calophyllum* species and their bioactive secondary metabolites, including calanolides, are already known [6,15,61,62,63,64,65,66,67]. Kudera et al. [66] reported in vitro growth inhibitory activity of *C. inophyllum* extract against diarrhea-causing microorganisms, e.g., *Clostridium difficile infant, Clostridium perfringens, Enterococcus faecalis, Escherichia coli, Listeria monocytogenes* and *Salmonella enterica*. The extract was particularly active against *C. perfringens* and *L. monocytogenes* (MIC = 128 μg/mL). Later, calanolide E (**10**) was isolated from *C. wallichianum* and tested for its anti-*Bacillus* activity against *Bacillus cereus, B. megaterium, B. pumilus* and *B. subtilis* [20]. However, calanolide E (**10**) was not bactericidal on the tested Bacillus species, and at the tested concentration. 

Based on the initial findings on promising antimicrobial properties of calanolides and *Calophyllum* extracts, efforts have recently been directed to the study on the effect of these compounds on the acid-fast bacillus *Mycobacterium tuberculosis* that causes tuberculosis [17,68,69]. As over the years several antibiotic resistant and multidrug-resistant *M. tuberculosis* strains have emerged, and complicated the existing treatment modalities for tuberculosis, and there has been a recent increase in incidents of tuberculosis globally observed, the need for new effective, safe and affordable antimycobacterial drugs has become paramount. *Calophyllum brasiliense* extract was reported to be active against *M. tuberculosis* (IC_50_ 3.02–3.64 μg/mL), and a follow up HPLC analysis of the active extract provided evidence of presence of calanolides and the antimycobacterial activity induced by *C. brasilliense* was attributed mainly to calanolides A (**1**) and B (**4**) [17]. Earlier, Xu et al. [68] demonstrated that calanolide A (**1**), from Colombian *C. lanigerum*, was active against both drug-susceptible and drug-resistant strains of *Mycobacterium tuberculosis,* e.g., H37Ra (ATCC 25177), H37Rv (ATCC 27294), CSU 19, CSU 33, H37Rv-INH-R (ATCC 35822), CSU 36, CSU 38 and H37Rv-EMB-R (ATCC 35837). Efficacy evaluations in macrophages established that this pyranocoumarin could inhibit intracellular replication of *M. tuberculosis* at concentrations below the minimum inhibitory concentration (MIC) determined in vitro. It was postulated that calanolide A (**1**), like the antitubercular drug rifampicin, could rapidly inhibit RNA and DNA synthesis followed by an inhibition of protein synthesis, and could lead to the generation of a new class of pyranocoumarin-based antitubercular drugs. In this study, the natural calanolides A (**1**), B (**4**) and D (**7**), as well as their semisynthetic analogues were tested, and (+)-calanolide A (**1**) and the semisynthetic analogue, 7,8-dihydrocalanolide B emerged as most effective against tuberculosis with the MIC value of 3.13 μg/mL. While (–)-calanolide B (**14**) was moderately effective, calanolide D (**7**) was found inactive at the highest tested concentration of 12.5 μg/mL. In fact, calanolides, especially calanolide A (**1**), is unique in a sense that these compounds have anti-HIV property and were found to be active against *M. tuberculosis* (MIC = 3.1 μg/mL) and an array of drug-resistant strains (MIC = 8–16 μg/mL). The antimycobacterial activity of calanolide A (**1**) is comparable to that of the well-known anti-tubercular drug isoniazid, and effective against rifampicin- and streptomycin-resistant *M. tuberculosis* strains. A recent patent described potent antimycobacterial property of calanolides and their analogs and provided a method of using these compounds for the treatment and prevention of mycobacterial infections [70].

### 4.4. Antiparasitic Activity

Traditionally, natural products, especially in crude forms, have long been used to treat various parasitic diseases, like babesiosis, leishmaniasis, malaria, trypanosomiasis and so on. Recently, leishmaniasis and trypanosomiasis have been in research focus of natural products researchers, aiming at discovering new drug candidates to treat these neglected diseases [71,72]. Extracts of *C. brasiliense* and *C. inophyllum* and calanolides were shown effective against intracellular parasites causing American trypanosomiasis and leishmaniasis [6]. In a recent study, Silva et al. (2020) [14] demonstrated that the MeOH extract from stem bark of *C. brasiliense* was active against amastigote forms of *Trypanosoma cruzi* and *Leishmania infantum*. Bioactivity-guided purification of the extra afforded calanolides E1 (**11**) and E2 (**12**), which were found to be active against *T. cruzi* (EC_50_ values of 12.1 and 8.2 μM, respectively) and *L. infantum*, (EC_50_ values of 37.1 and 29.1 μM, respectively) in vitro. Calanolide E1 (**11**) displayed the best selectivity index (SI) with values >24.4 to *T. cruzi* and >6.9 to *L. infantum* in comparison to calanolide E2 (**12**). It was concluded that these coumarins could be utilized as scaffolds for the design and development of novel drug candidates to treat Leishmaniasis and Chagas diseases.

## 5. Toxicological Aspects Including Pharmacokinetics

Among the naturally occurring calanolides (Figure 2), calanolide A (**1**), a specific nonnucleoside inhibitor of human immunodeficiency virus type 1 (HIV-1) reverse transcriptase, first isolated from a tropical tree *C. lanigerum* that grows abundantly in the Malaysian rain forest, is the most-studied compound in terms of its pharmacology, toxicology and synthesis. A series of animal studies [43] involving mice, rats and dogs established that calanolide A (**1**) is generally well-tolerated at oral doses of up to 150 mg/kg in rats and 100 mg/kg in dogs, and possesses a good safety profile [73,74]. Calenolides A (**1**), B (**4**) and C (**6**) were found to be nontoxic in mice (LD_50_ = 1.99 g/kg), and no alternation on hepatocytes could be observed during the histological study of the mice treated with the highest dose applied [74]. During a study looking at the anti-HIV efficacy and toxicity of calanolides when used in combination with other anti-HIV drugs, no noticeable toxicity could be detected [46]. 

In the very first study on the safety and pharmacokinetics of calanolide A (**1**) in healthy HIV-negative human volunteers revealed that the toxicity of calanolide A (**1**) was minimal in the majority of subjects treated with four successive single dose, 200, 400, 600 and 800 mg. While there were no acute serious or life-threatening adverse effects were observed, among the usual minor adverse effects, dizziness, oily taste, headache, eructation, and nausea were noticed, but were of minimal clinical significance. These adverse effects were non-dose-dependent [73]. In this study, it was found that calanolide A (**1**) was rapidly absorbed following administration, with time to maximum concentration of drug in plasma (*T*_max_) values, depending on the doses, occurring between 2.4 and 5.2 h. It was noted that the levels of calanolide A (**1**) in human plasma were higher than would have been predicted from animal studies, but the safety profile was benign. However, taking calanolide A (**1**) with food was found to generate significant variability in pharmacokinetics, but with no detectable interaction with food. Later, these findings were further confirmed by another similar study carried out by Eiznhamer et al. [75]. Calanolide A (**1**), the first member of the new family of NNRTIs, was found to have long elimination half-life, the benign toxicity profile, to achieve trough plasma levels approximating the EC_90_ of calanolide A (**1**) for HIV-1, to have the potential for twice daily dosing, and to offer the unique HIV-1 resistance profile could make this compound an attractive candidate for further clinical studies. It was reported that after oral administration, (+)-calanolide A (**1**) was generally well tolerated and indication of any safety concern could be observed [48]. Its plasma concentrations in humans were higher than anticipated from animal data. The AUC and C_max_ values increased with increasing dose, and it appeared that therapeutic levels could easily be achieved in humans. 

A comparative study on the relative pharamacokinetic parameters and bioavailability of calanolide A (**1**) and its synthetic analogue dihydrocalanolide A (**15**) was reported [76]. This study compared the intravenous pharmacokinetics of the dihydro analog relative to the parent compound, calanolide A (**1**), and determined the relative oral bioavailability of each drug in CD2F1 mice. Both compounds displayed similar pharmacokinetic parameters, but the oral bioavailability of the dihydro analogue was considerably better (almost 3.5-fold) than calanolide A (**1**). Moreover, the relative ability of calanolide A (**1**) and its dihydro analog to change to their inactive epimer forms, (+)-calanolide B (**4**) and (+)-dihydrocalanolide B, respectively, was also determined; while conversion of active calanolides to inactive forms occurred in vitro especially under acidic conditions, no epimers of either compound were observed in plasma of mice after administration of either (+)-calanolide A (**1**) or (+)-dihydrocalanolide A (**15**). It was suggested that the selection of the dihydro derivative of calanolide A (**1**) could be a reasonable choice for further preclinical development and possible Phase I clinical evaluation as an oral drug candidate for the treatment of HIV infection. Calanolide A (**1**) was shown to be distributed readily into the brain and lymph [55]. The distribution and elimination pattern of calanolide A (**1**) and its 7,8-dihydro derivative were found to be similar, but the apparent volume of distribution (Vd) and oral clearance of these compounds were significantly different after oral administration. It was also demonstrated that calanolide A (**1**) is generally well tolerated in doses up to 600 mg. As evident from animal studies, the gastrointestinal intolerance for this compound is not severe, but the most common adverse events as observed in human trails of calanolide A (**1**) include an oily after taste and transient dizziness [55]. The calculated half-life of calanolide A (**1**) from 800 mg dosing was reported to be 20 h [55,73]. 

During the study directed to the evaluation of antitubercular property of calanolides and their semisynthetic analogues, the pharmacokinetic data indicated that the (+)-calanolide A (**1**) concentrations in plasma could be comparable to the observed in vitro MICs against *M. tuberculosis* [68]. Both calanolides A (**1**) and B (**4**) metabolized by cytochrome P450 CYP3A, and their blood levels could be enhanced if co-administered with ritonavir. Usach et al. [77] reported the safety, tolerability and pharmacokinetics profiles of calanolide A (**1**), as a result of a comprehensive Phase I clinical trial.

## 6. Therapeutic Potential

Naturally occurring calanolides and their synthetic or semi-synthetic analogs have undergone several pre-clinical and clinical trials for their anti-HIV activity, aiming at novel anti-HIV drug development [2,16,55,78]. In fact, calanolide A (**1**) was at an advanced stage of development as an anti-HIV drug about a decade ago [78]. Buckheit [79] reviewed therapeutic potential of non-nucleoside reverse transcriptase inhibitors like calanolides as anti-HIV and commented on strategies for the treatment modalities for HIV infections. In fact, NNRTIs opened a new avenue of treatment of HIV infections, as previously this therapeutic area was predominantly covered by nucleoside reverse transcriptase inhibitors and protease inhibitors. Soon after the discovery of calanolides as a potential ant-HIV agents by the NCI/NIH, Sarawak Medichem Pharmaceuticals (Sarawak, Malaysia) synthesized calanolide A (**1**) and started developing calanolide A (**1**) as a clinical drug for the treatment of HIV infections. It was a joint venture between the Sarawak State Government and Medichem Research Inc. 

During 2001–2005, an interventional clinical trial was conducted on human volunteers [80], where patients were randomized to receive (+)-calanolide A (**1**) or placebo for 21 days. All patients could elect to receive an open-label, 3-month course of approved retroviral therapy (up to triple-drug therapy) to be selected by, and administered under the care of, the patients’ physicians. If the patient had no insurance coverage or did not wish to utilize his/her insurance for anti-HIV medications, Sarawak MediChem Pharmaceuticals provided these medications at no charge for up to three months. The trial was primarily aimed at the assessment of the safety and effectiveness of (+)-calanolide A (**1**) in HIV-infected patients who had never taken anti-HIV drugs. In 2006, Craun Research (Kuching, Malaysia), a company established by the Sarawak Government, acquired Sarawak MediChem, and in 2016, Craun Research announced the completion of Phase I clinical trials for calanolide A (**1**) with doses of 200 to 800 mg, which initially started in 2013 [77]. In 2017, F18 (10-chloromethyl-11-demethyl-12-oxo-calanolide A), a synthetic structural analog of calanolide A (**1**) was shown to have more potent anti-HIV activity than original molecule, calanolide A (**1**) [81,82]. This compound showed better druggable profile with 32.7% oral bioavailability in rat, tolerable oral single-dose toxicity in mice, and suppressed both the wild type HIV-1 and Y181C mutant HIV-1 at an EC_50_ of 7.4 nM and 0.46 nM, respectively [83]. Furthermore, it was shown that two enantiomers F18, (*R*)-F18 and (*S*)-F18, had quite similar anti-HIV property, but (*R*)-F18 was more potent than (*S*)-F18 against wild type virus, K101E mutation and P225H mutation pseudoviruses [81]. However, calanolides, particularly calanolide A (**1**) remains as an investigational anti-HIV drug and has not yet been approved by the FDA or any other drugs regulatory bodies for their commercial pharmaceutical production.

## 7. Patents

In 1999, calanolides and related antiviral compounds were patented by the Board of Trustees of the University of Illinois [84]. The patent covered novel antiviral compounds, calanolides, and their derivatives that could be isolated from plants of the genus *Calophyllum* in accordance with the specified method. The patent also included the uses of these compounds and their derivatives alone or in combination with other antiviral agents in compositions, such as pharmaceutical compositions, to inhibit the growth or replication of a virus, such as a retrovirus, in particular a human immunodeficiency virus, specifically HIV-1 or HIV-2. Later, another patent, owned by Parker Hughes Institute, was reported, which described the novel uses of calanolides as Tec family/BTK (Bruton’s tyrosine kinase) inhibitors, methods for their identification, and pharmaceutical compositions [85]. It can be mentioned here that the BTK inhibitors inhibit the enzyme BTK, which is a crucial part of the B-cell receptor signaling pathway, and these inhibitors have emerged as a new therapeutic target in a variety of malignancies, e.g., chronic lymphocytic leukemia and small lymphocytic lymphoma [86].

## 8. Conclusions

Non-nucleoside reverse transcriptase inhibitors (NNRTIs), efavirenz, nevirapine and delavirdine, have become one of the cornerstones of highly active anti-retroviral therapy for HIV infections. Calanolides, as they belong to this pharmacological class of NNRTIs, and because of their high safety margins and favorable pharmacokinetic profiles, are ideal candidates for novel anti-HIV drug development. While several analogues of the naturally occurring calanolides have been synthesized, a good number of preclinical and clinical trials have been conducted to date, and there are a few patents published, further work is still required to commercially bring any of the calanolide candidates, natural or synthetic, to anti-HIV drug market. As calanolides show an excellent synergistic and additive profile in combination with other anti-HIV drugs, it is assumed that calanolides can be considered for use in combination therapy for HIV infections.

## Figures and Tables

**Table 1:** Naturally occurring calanolides, their sources and properties.

Calanolides	Sources	Physical State	Mol. Formula	Mol. Weight	Optical Rotation [a]_D_	UV λ_max_ (MeOH) nm	References
Calanolide A (**1**)	*Calophyllum lanigerum var. austrocoriaceum *	Oil	C_22_H_26_O_5_	370.44	[a]_D_ +60° (c, 0.7 in CHCl_3_)	228, 284 and 325	[1,9,11,16]
	*Calophyllum brasiliense *						[4,17,18]
	*Calophyllum inophyllum *						[6,11]
	*Calophyllum teysmannii *						[19]
	*Clausena excavata *						[10]
12-*O*-Acetylcalanolide A (**2**)	*Calophyllum lanigerum var. austrocoriaceum *	Oil	C_24_H_28_O_6_	412.48	[a]_D_ +20° (c, 0.5 in CHCl_3_)	228, 284 and 325	[1]
12-*O*-Methylcalanolide A (**3**)	*Calophyllum lanigerum var. austrocoriaceum *	Oil	C_23_H_28_O_5_	384.47	[a]_D_ +32° (c, 0.8 in CHCl_3_)	228, 284 and 325	[1]
Calanolide B (**4**)	*Calophyllum lanigerum var. austrocoriaceum *	Oil	C_22_H_26_O_5_	370.44	[a]_D_ +8° (c, 1.0 in acetone)	228, 284 and 325	[1]
	*Calophyllum brasiliense *						[5,17]
	*Calophyllum teysmannii var. inophylloide *						[9]
12-*O*-Methyl-calanolide B (**5**)	*Calophyllum lanigerum var. austrocoriaceum *	Oil	C_23_H_28_O_5_	384.47	[a]_D_ +34° (c, 0.5 in CHCl_3_)	228, 284 and 325	[1]
Calanolide C (**6**)	*Calophyllum brasiliense*	Oil	C_22_H_26_O_5_	370.44	-	-	[4,5]
Calanolide D (**7**)	*Calophyllum brasiliense*	Amorphous solid	C_22_H_24_O_5_	368.42	-	-	[6]
Calanolide E (**10**)	*Calophyllum lanigerum var. austrocoriaceum *	Amorphous powder	C_22_H_28_O_6_	388.50	[a]_D_ +30° (c, 0.7 in acetone)	-	[1,9,20]
	*Calophyllum membranaceum *						[21]
	*Calophyllum molle *						[9]
	*Calophyllum polyanthum *						[22]
	*Calophyllum teysmannii var. inophylloide *						[20]
	*Calophyllum wallichianum*						[15]
Calanolide E1 (**11**)	*Calophyllum lanigerum var. austrocoriaceum*	Amorphous powder	C_22_H_28_O_6_	388.50	-	-	[9,20]
	*Calophyllum brasiliense*						[9,14]
	*Calophyllum molle*						[9]
Calanolide E2 (**12**)	*Calophyllum lanigerum var. austrocoriaceum*	Amorphous powder	C_22_H_28_O_6_	388.50	-	-	[9,20]
	*Calophyllum brasiliense* Cambess.						[14]
	*Calophyllum membranaceum*						[21]
	*Calophyllum molle*						[9]
	*Calophyllum polyanthum*						[22,23]
	*Calophyllum teysmannii var. inophylloide*						[9,20]
Calanolide F (**13**)	*Calophyllum lanigerum var. austrocoriaceum*	Amorphous powder	C_22_H_26_O_5_	370.44	[a]_D_ −51.5 (c, 0.3 in CHCl_3_)	227, 283, 322	[9,20]
	*Calophyllum teysmannii var. inophylloide*						[9,20]
Costatolide (**14**) [(−)-Calanolide B]	*Calophyllum brasiliense*	Crystals (M.p. 181–183°)	C_22_H_26_O_5_	370.44	[a]_D_ −19.9 (c, 0.42 in CHCl_3_)	228, 284 and 325	[4]
	*Calophyllum costatum*						[24]
	*Calophyllum inophyllum* L.						[24]
	*Calophyllum teysmannii var. inophylloide*						[9,19,25]
7,8-Dihydrocalanolide A (**15**)	*Calophyllum lanigerum var. austrocoriaceum*	Amorphous solid	C_22_H_28_O_5_	372.46	Negative optical rotation	-	[25]
Dihydrocostatolide (**16**)	*Calophyllum costatum*	Amorphous solid	C_22_H_28_O_5_	372.46	-	-	[26]
Pseudocalanolide C (**8**) [incorrectly named as calanolide C (**6**)]	*Calophyllum lanigerum var. austrocoriaceum*	Amorphous solid	C_22_H_26_O_5_	370.44	[a]_D_ +68° (c, 0.7 in CHCl_3_)	-	[1,3,9]
Pseudocalanolide D (**9**) [incorrectly named as calanolide D (**7**)]	*Calophyllum lanigerum var. austrocoriaceum*	Amorphous solid	C_22_H_24_O_5_	368.43	[a]_D_ +60° (c, 0.5 in CHCl_3_)	-	[1,3]
Tomentolide B (**17**)	*Calophyllum tomentosa*	Amorphous solid (M.p. 158–160^o^)	C_22_H_24_O_5_	368.43	Racemic mixture	-	[1,3,9]
